# Supplementary material for: Identification of photoperiod-induced specific miRNAs in the adrenal glands of Sunite sheep (Ovis aries)
Source: Front Vet Sci. 2022 Jul 22;9:888207. doi: 10.3389/fvets.2022.888207 (PMC9354845; doi:10.3389/fvets.2022.888207)
Supplement: Supplementary file 1 [file Data_Sheet_1.ZIP › Supplementary file/miRDeep2 file.zip!/miRDeep2 file/Novel_Structure/LP42A3/Novel_317.pdf]

Provisional ID	:	NC_019460.2_4931
Score total	:	1.4
Score for star read(s)	:	-1.3
Score for read counts	:	0
Score for mfe	:	1.1
Score for randfold	:	1.6
Score for cons. seed	:	
Total read count	:	133
Mature read count	:	133
Loop read count	:	0
Star read count	:	0

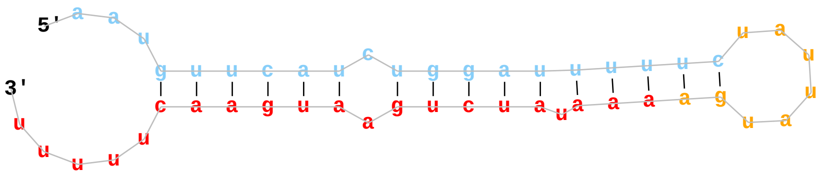

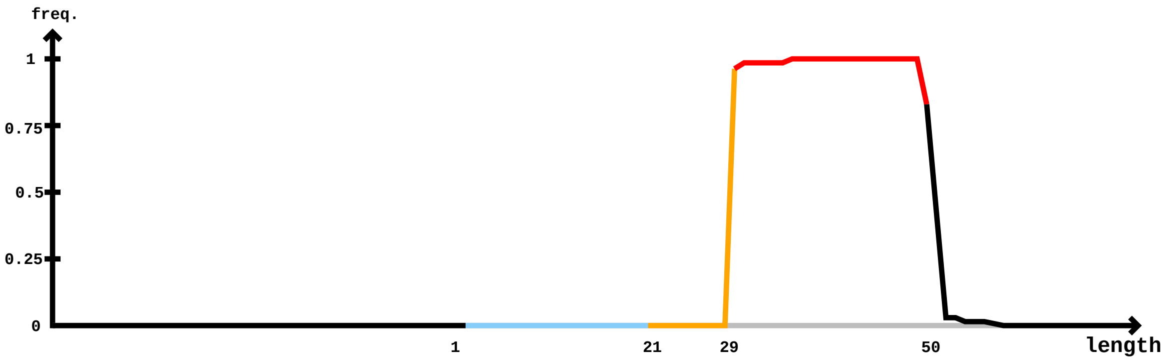

Star Mature

```
5'- aagaaauauuuucaaaaauauuuuaauuuuaaguuggccaaaauguucaucuggauuuuucuauuaugaaaauaucugaaugaacuuuuuggccaaucaaauacagguuuc   -3'    exp
    ..(((((((((....)))))))))........(((((((((((.((((((.((((((((((......))))).))))).)))))).))))))))))).............    reads      mm         sample
    .....................................................................aaaAaucugaaugaacuuuu......................    23         1          seq
    .....................................................................aaaAaucugaaugaacuuuuu.....................    65         1          seq
    .....................................................................aaaAaucugaaugaacuuuuug....................    42         1          seq
    ......................................................................aauaucugaaugaacuuuuug....................    1          0          seq
    ......................................................................aaAaucugaaugaacuuuuuggc..................    2          1          seq
    ...........................................................................cugaaugaacuuuuuUgccaa...............    1          1          seq
    ...........................................................................cugaaugaacuuuuugAccaau..............    1          1          seq
```
